# Carbon‐13 Centerband‐Only Detection of EXchange with Dynamic Nuclear Polarization

**DOI:** 10.1002/cphc.202500585

**Published:** 2026-01-30

**Authors:** Abel Cherian Varkey, Kai Xue, Evgeny Nimerovsky, Stefan Becker, Loren B. Andreas

**Affiliations:** ^1^ Department of NMR Based Structural Biology Max Planck Institute for Multidisciplinary Sciences Am Fassberg 11 37077 Göttingen Germany; ^2^ NTU Centre of High Field NMR Spectroscopy and Imaging Nanyang Technological University 21 Nanyang Link Singapore 637371 Singapore; ^3^ School of Physical and Mathematical Science Nanyang Technological University 21 Nanyang Link Singapore 637371 Singapore

**Keywords:** Centerband‐Only Detection of EXchange, dynamic nuclear polarization, magic‐angle spinning, oligomers, phenylalanine

## Abstract

The magic‐angle spinning NMR technique, Centerband‐Only Detection of EXchange (CODEX), can be used to determine the oligomerization state of molecules when combined with site‐specific labeling. Calibrated with amino acid crystals, the method is successfully applied to proteins, primarily combined with ^19^F labeling. The use of ^13^C spins for CODEX‐based oligomer determination in proteins is hampered by limited sensitivity of ^13^C spins due to the low gyromagnetic ratio of ^13^C and the presence of natural abundance background spins which contribute to the observed CODEX decay. The use of CODEX is proposed in conjunction with dynamic nuclear polarization (DNP) at low temperature to increase sensitivity. It is necessary to correct for effects of ^13^C present at natural abundance. A (PDSD) proton driven spin diffusion‐based correction is demonstrated to be effective when the isotropic chemical shifts of the natural abundance background are distinct from the labeled site. Using a ^13^C‐*ζ*‐phenylalanine‐labeled GB1 sample, it is demonstrated that the autocorrelation peak decay observed in a series of PDSD spectra can be utilized to correct for the additional dephasing and recover the expected CODEX decay curve. With ^13^C‐*γ*‐phenylalanine labeling and ^13^C‐depleted background, mixing times up to 1500 s are demonstrated.

## Graphical abstract

A combination of dynamic nuclear polarization‐based signal enhancement and cryogenic conditions enables counting of spins in a cluster in proteins using ^13^C‐Centerband‐Only Detection of EXchange when combined with background correction. Optionally, ^13^C‐depleted labeling of background spins further improves the method.

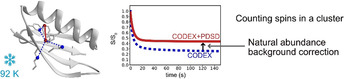

## 1 Introduction

**Figure 1:**
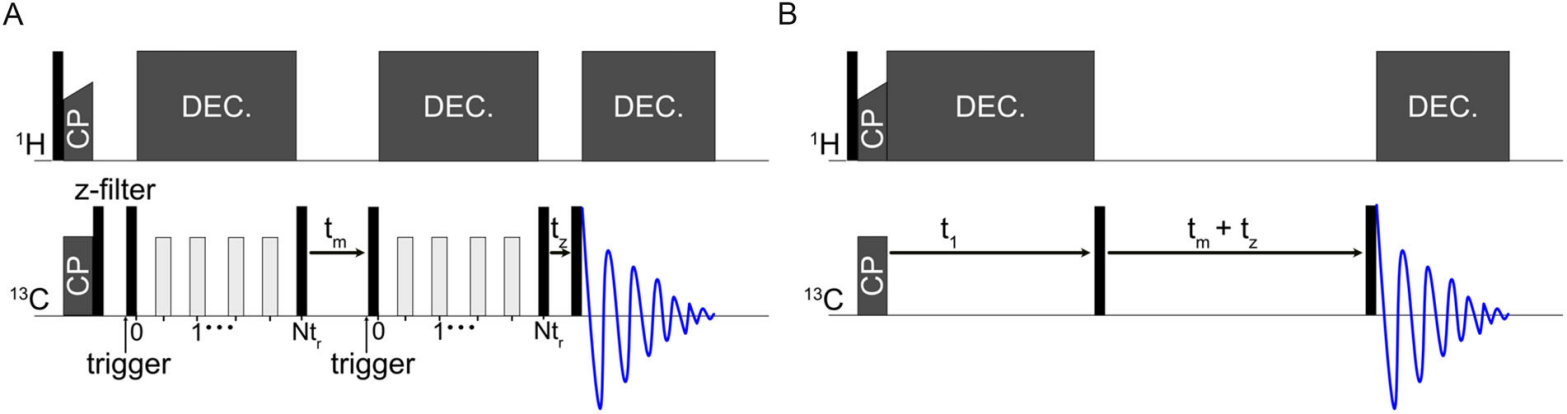
A) CODEX pulse sequence. The black rectangles represent 90° pulses, and the gray rectangles represent 180° pulses. *t*_m_ is the mixing period, and *t*_z_ is a z‐filter. For the reference experiment, the length of *t*_m_ and *t*_z_ is exchanged, which compensates for *T*_1_ relaxation. Dec. represents high‐power SPINAL64 decoupling. B) PDSD pulse sequence.

Centerband‐Only‐Detection‐of‐Exchange (CODEX) is a technique originally developed for measuring molecular motion in the timescale of milliseconds to seconds, slower than the inverse of the magnitude of the chemical shift anisotropy (CSA).^[^ ^1^, ^2^ ^]^ The principle of the experiment is briefly described below, and the pulse sequence is depicted in **Figure** **1**A. Following a 90‐degree pulse, a series of rotor‐synchronized 180° pulses recouples the CSA, such that phase is acquired dependent on molecular orientation. Subsequently, a 90° pulse flips one of the transverse components of the spin to the z‐axis. After a mixing period, another 90° pulse flips the magnetization vector into the transverse plane, and after another block of CSA recoupling pulses, the stimulated echo is recorded. The first set of recoupling pulses is called the encoding period, and the second set the decoding period. If no change occurs during the mixing period, a full stimulated echo is observed. A change in the CSA Hamiltonian in the intervening mixing period, either due to molecular reorientation or spin diffusion to a neighboring spin with a distinct CSA or a different CSA orientation, prevents the signal from refocusing.

CODEX has been implemented numerous times for the study of molecular motion in various systems ranging from materials to biological systems. Examples include the study of residual acetate distribution and kinetics of exchange with water in a metal–organic framework (MOF) probed with ^13^C CODEX,^[^ ^3^ ^]^ investigation of the dynamics in polyrotaxanes formed from the capped supramolecular complex of polyethylene glycol (PEG) and cyclodextrin allowed an atomistic description of slow dynamics for different components of the complex,^[^ ^4^ ^]^ and the impact on phospholipid diffusion in a lipid bilayer upon cationic peptide addition using ^31^P CODEX.^[^ ^5^ ^]^

Taking advantage of spin diffusion, rather than dynamic reorientation of the CSA, CODEX has been cleverly repurposed for the determination of oligomer states in samples where motion on slow time scales is not significant.^[^ ^6^, ^7^ ^]^ During the mixing period, spin diffusion causes equilibration of spin polarization among nearby spins. The signal at long mixing time points will decay to the reciprocal of the total number of orientationally inequivalent spins, including spins with identical CSA parameters. The distances up to which spin diffusion can occur are often limited by *T*_1_ relaxation of the starting magnetization.

Hong and coworkers estimated spin diffusion rates from CODEX curves based on known distances in various amino acid crystals with single‐site labels which included glycine, leucine, phenylalanine, and tryptophan.^[^ ^7^, ^8^ ^]^ This provided the basis for ^19^F CODEX studies of membrane proteins, such as matrix 2 protein (M2) from Influenza A virus,^[^ ^7^ ^]^ gp41 from HIV,^[^ ^9^ ^]^ and the Envelope protein from SARS‐CoV‐2.^[^ ^10^, ^11^ ^]^ The large gyromagnetic ratio of ^19^F allows for distance measurements beyond 1 nm.^[^ ^8^ ^]^

CODEX for oligomer determination has often been implemented with a single‐site label per molecule, such that the signal ultimately decays to the inverse of the oligomer number, provided that the labeled sites are in close enough proximity for spin diffusion transfer. Since CODEX for oligomer investigation is based on broad‐band spin diffusion, the presence of any “contaminating” spins, such as the 1.1% natural abundance of ^13^C, contributes to the signal decay curve. This makes ^19^F labeling, which is essentially background‐free in biological samples, particularly attractive. Furthermore, ^19^F has a high gyromagnetic ratio which aids spin diffusion over greater distances compared to ^13^C and ^15^N. For these reasons, ^19^F NMR is gaining in popularity. Nevertheless, due to the relative ease of introducing ^13^C labels, as compared to ^19^F into recombinantly expressed proteins,^[^ ^12^ ^]^ the application of ^13^C CODEX would become a desirable alternative if the challenges of sensitivity and natural abundance spins could be overcome.

In an earlier report, the problem of natural abundance background ^13^C spins was addressed by estimating the probability of a labeled site encountering an unlabeled site.^[^ ^13^ ^]^ It was necessary to include this correction factor for the accurate determination of the number of molecules in an expanded unit cell of phenylalanine crystals comprising eight molecules.^[^ ^14^ ^]^

This correction factor requires prior knowledge of the spin density within a volume element where spin diffusion can occur. If the number of spins per unit volume is not known, then spins from unlabeled sites will contribute to an unknown degree. For example, a sphere of 10 Å radius around an atom located in the core of the immunoglobulin‐binding B1 domain of streptococcal protein G (GB1), Protein Data Bank (PDB) code 00002QMT, contains 133 carbon atoms.^[^ ^15^ ^]^ For a pair of spins separated by 6.4 Å, this number grows to 172 carbon atoms. At 1.1% natural abundance, this translates to about 1.9 additional contributing spins on average. Therefore, a complex model that includes the number of natural abundance spins is crucial to distinguish between dephasing due to chemically equivalent and chemically inequivalent spins. This calls for a method to correct for the additional CODEX decay that occurs due to nuclear spins present at unlabeled sites without having prior knowledge of the spin density of the additional spins.

The distance over which ^13^C spin diffusion occurs is also limited by a lower gyromagnetic ratio than ^19^F, and thus, all else being equal, the intermonomer distance in oligomers would have to be shorter as compared with ^19^F CODEX. This limitation can be partially overcome by increasing the signal with the hyperpolarization technique DNP NMR.^[^ ^16^, ^17^ ^]^ DNP NMR is well established for biological systems and typically entails addition of a biradical polarizing agent in a glass‐forming matrix, such as glycerol–water. Microwave irradiation and the cross‐effect mechanism lead to hyperpolarization of nuclear spins. The optimization of ^1^H hyperpolarization allows for fast repetition of the experiment, since proton spin diffusion spreads the signal across the sample through the strong homonuclear dipolar coupling network, providing the source polarization for cross‐polarization to ^13^C or ^15^N (or other nuclei) for detection.^[^ ^18^ ^]^ A typical DNP signal enhancement factor is 10–30 for biological samples and can be in the 100s for well‐behaved samples.^[^ ^19^ ^]^ It was previously shown for carbonyl‐labeled Phe crystals at 8 kHz MAS that the upper limit for ^13^C spin diffusion at 100 K is above 9 Å but below about 12.5 Å.^[^ ^13^ ^]^ Thus, with the DNP enhancement, measurements at 100 K should allow extension to moderately sized proteins. Furthermore, molecular motion on a slow timescale which can potentially interfere with the experiment is frozen out at low temperatures.^[^ ^20^ ^]^

In this report, we show for the model protein GB1 that ^13^C‐based CODEX data can be corrected using a separate 2D correlation experiment that accounts for the additional decay contributed by the natural abundance of ^13^C at sites that are resolved in chemical shift. The use of DNP signal enhancement allows spin diffusion times of several minutes, corresponding to a distance range of about 1 nm where this correction is needed.

## 2 Theoretical Background

During the mixing block of a CODEX experiment, spin polarization equilibrates between neighboring spins via spin diffusion. In an ideal sample with single‐site labels, the spins exchange spin polarization at a rate determined by their relative distances and orientations, providing valuable information about oligomer number and intermonomer distances. Since the labeled sites are chemically equivalent, the decay in the peak intensity in such a CODEX measurement is due to the different CSA orientations as well as *T*_1_ relaxation. CODEX experiments usually involve a separate reference experiment, in which a short z‐mixing period is placed between the recoupling blocks, while the longer spin diffusion period is placed after the second CSA recoupling block to account for the relaxation under *T*_1_ for the same length of time as the corresponding dephased spectrum. This permits the disambiguation of dephasing from relaxation decay to give a pure exchange curve.^[^ ^1^ ^]^

The intensity of the CODEX dephased spectrum, *S*, is reduced due to exchange with neighboring spins and is typically plotted as a function of the mixing time (*t*_m_). The resulting CODEX curve decays due to spin diffusion to other chemically equivalent labeled sites with different orientations, but also to spins with a different isotropic chemical shift (ICS). Natural abundance ^13^C spins are randomly populated in a volume element around the labeled spins. As the volume over which spin diffusion occurs increases with time, the number of relevant background spins also increases. Thus, the curve does not reach equilibrium until the signal is equilibrated over the size of the molecular system. For extended biological systems without an ordered lattice, a more practical limit is often reached when there is insufficient signal due to *T*_1_ relaxation processes.

The CODEX reference spectrum, *S*_0_, on the other hand, is assumed to only decay with *T*_1_ relaxation. It preserves signal intensity due to negligible anisotropic and isotropic exchange during the short mixing time (*t*_z_) placed between the encoding and decoding blocks. Subsequently, during *t*_m_, any signal that arises due to exchange with chemically identical sites does not result in decay since the sites have the same ICS. Exchange from and to sites at natural abundance (with a different ICS) can be expected to be in balance, such that the intensity is not impacted, provided that the *T*_1_ relaxation times of the sites are similar.

Comparing the CODEX dephased spectrum to the reference spectrum, therefore, will not be a pure readout of the labeled sites with different orientations, rather it will contain contributions from exchange to other sites with a different ICS.

To isolate the effect of exchange to sites with a different ICS, we propose using a 2D ^13^C–^13^C correlation spectrum with the same PDSD^[^ ^21^ ^,^ ^22^ ^]^ mixing as applied during CODEX. The autocorrelation peaks (both diagonal and off‐diagonal peaks in the sideband manifold) of the site of interest will decay in the PDSD spectrum due to *T*_1_, but also due to exchange to sites with a different ICS. It therefore provides a more relevant correction than the *S*_0_ curve, since it not only accounts for *T*_1_ relaxation, but also exchange to sites with different ICS:

(1) $$I_{\text{corr}}\left(t\right)=\frac{S\left(t\right)}{CC\left(t\right)}\times \frac{CC\left(0\right)}{S\left(0\right)}$$

where $I_{\text{corr}}$ is the corrected CODEX decay, S(t) is the CODEX decay spectrum, and *CC(t)* is the intensity of the autocorrelation peaks of the labeled site. The term, $\frac{CC\left(0\right)}{S_{0}\left(0\right)}$, allows for a comparison of the 2D intensity with the 1D intensity. Note that S(0) is equivalent to $S_{0}\left(0\right)$, and that in practice, a short delay is used rather than recording with zero mixing, which reduces demands on the phase cycle. It is assumed that no significant mixing occurs for this short delay.

Thus, the standard 1D CODEX reference is not needed, and time points are referenced to the autocorrelation peak intensity of the 2D PDSD spectrum. In practice, we have acquired all the CODEX reference spectra as a point of comparison. The approach is conceptually similar to the 2D CODEX introduced by Mei Hong and coworkers, but requires only half the number of 2D spectra.^[^ ^23^ ^]^

## 3 Results and Discussion

We demonstrate the applicability of this correction factor for CODEX measurements on a frozen solution of ^13^C‐*ζ*‐phenylalanine‐labeled GB1. Two phenylalanine residues are present in GB1, within the hydrophobic core of the protein. The distance between the two C*ζ* sites of the phenylalanine residues is ≈6 Å (PDB: 00002QMT), well within the expected range of spin diffusion. The resulting two‐site labeling in the protein core implies that the CODEX curve should decay to 50%, assuming that no peaks overlap with the labeled site.

**Figure 2:**
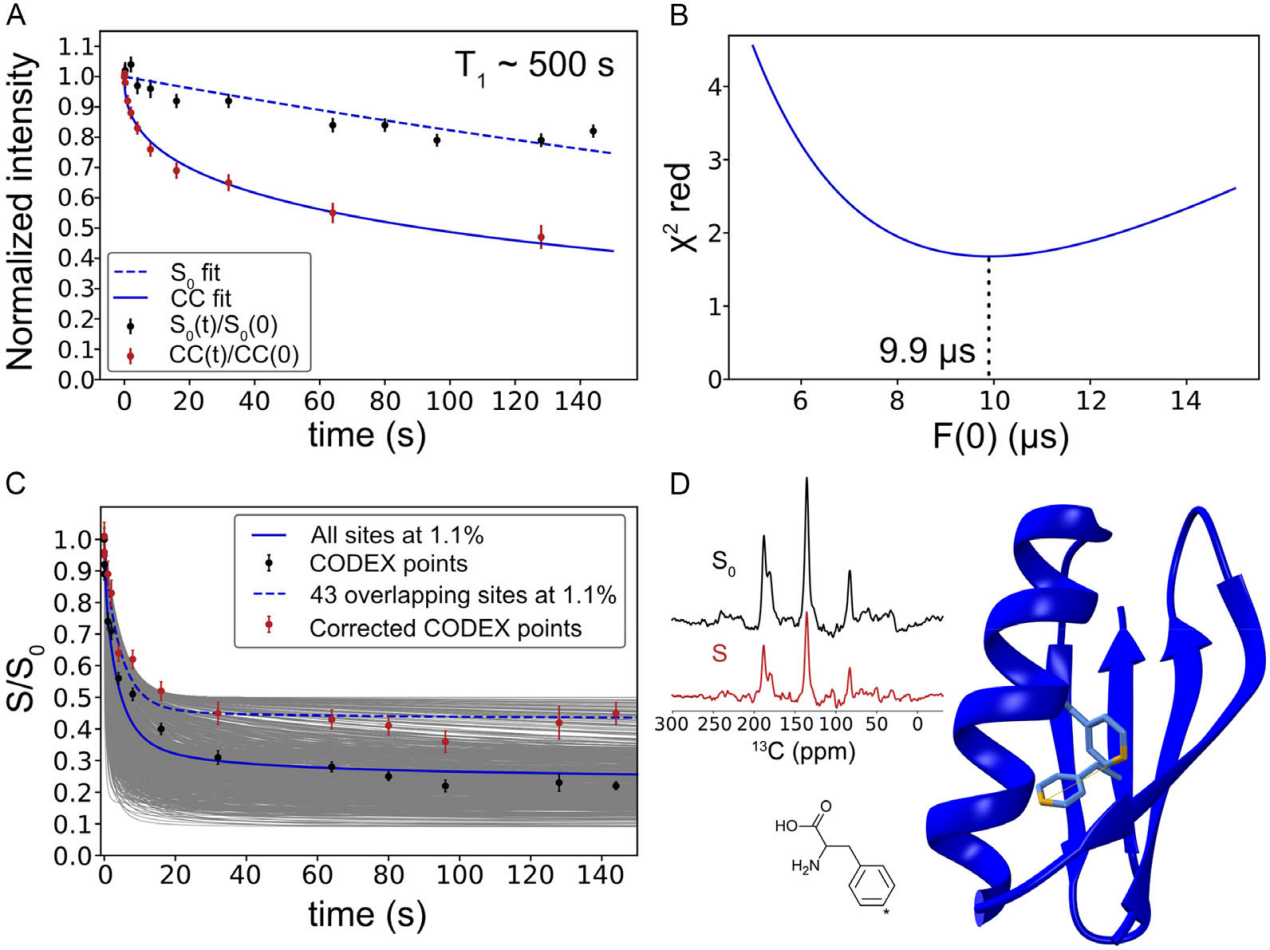
^13^C CODEX of Phe C*ζ*‐labeled GB1 A) Normalized reference curves: 1D S_0_ and 2D autocorrelation intensities. The *S*_0_ decay is fit to an exponential function and the PDSD decay is fit to a stretched exponential function. The *T*_1_ is estimated from the *S*_0_ curve. B) The reduced chi‐square of the fit to the corrected CODEX points in C. C) Experimental data and simulated curves with consideration of the natural abundance of ^13^C. The gray lines display individual runs of the simulation for the case where all carbon sites were populated according to natural abundance background of 1.1%. The solid blue line shows the ensemble average of these individual simulation runs. The dashed blue line shows a simulated curve of Phe C*ζ*, including unlabeled sites that overlap with the labeled site ICS. D) Example CODEX spectrum recorded with 2 s of mixing, and the structure of GB1 (PDB: 00002QMT) with the ^13^C‐labeled C*ζ* site marked in gold in the protein structure. The C*ζ* position on the Phe ring is highlighted with an asterisk.

In the PDSD spectrum, the C*ζ* autocorrelation peak decays in intensity due to the effect of spin diffusion and *T*_1_, as mentioned earlier (**Figure** **2**A). This decay is fit to a stretched exponential function of the form $CC\left(t\right)=CC\left(0\right)\times e^{{\text{‐t}}^{\beta }\text{/T}}$, and this fit curve is used to correct the CODEX curve. The values obtained for *T* and *β* are 11.06 s and 0.46, respectively. Correcting the CODEX curve with the fit avoids the need to record PDSD spectra for all CODEX time points, and also averages the scatter in the points, thus giving a more uniform correction. The substantial difference between this decay curve and decay under *T*_1_ relaxation highlights the significant loss of signal via spin diffusion to natural abundance ^13^C sites in the protein, which have a different ICS.

Note that the ^13^C *T*_1_ under DNP conditions is at least two orders of magnitude longer than the ^19^F *T*_1_ of ^19^F‐Phe‐labeled biomolecules measured near room temperature.^[^ ^10^ ^]^ This facilitates the measurements at long mixing times of over 100 s, which is needed to equilibrate the CODEX curve for the relatively long distance of about 6 Å. Considering that the spin diffusion rate scales with the fourth power of the gyromagnetic ratio, around 200‐fold longer CODEX mixing is needed for ^13^C versus ^19^F for measurement of similar distances (assuming similar *F*(0) values).

Figure 2B shows a fit to determine the F(0) value, which scales the spin diffusion rate, and depends on multiple factors, such as spinning frequency and chemical shift tensors, as explained further in the Experimental Section. The F(0) value was determined to be 9.9 μs with a reduced χ^2^ value of 1.68 by fitting a simulated curve to the corrected CODEX curve with overlapping sites considered, as explained below. This F(0) value was used for all the subsequent simulation runs.

Figure 2C shows both corrected and uncorrected CODEX curves, together with simulated curves that consider the presence of background spins. The 1000 gray curves show each individual run with natural abundance ^13^C spins randomly populated at 1.1% probability across all carbon atoms in the protein (excluding the labeled sites). There are 272 such sites, and each individual simulation had between two and ten spins. The solid blue line shows the average of these natural abundance curves, which decays to around 0.25 at 150 s and fits to the uncorrected data displayed as black points. This is significantly lower than the expected 0.5 when considering only labeled sites, showing that correction is necessary for the longer CODEX mixing times of ≈30 to 150 s.

A limitation of the correction is the inability to account for spins that lie within the linewidth of the ICS of the labeled site. This is expected, since spin diffusion to these sites is retained in the autocorrelation peaks in the 2D PDSD spectrum. Therefore, these sites must be accounted for based on prior knowledge of the spins at that frequency in the protein sample. The C*ζ*‐labeled Phe peak in GB1 appears at 129.2 ppm at 95 K with a linewidth of ≈5 ppm. From the BMRB chemical shift database, the potential overlapping resonances are C*δ*1/2 and C*ε*1/2 from Phe, C*δ*1 and C*δ*2 from Trp, and C*γ* and C*δ*1/2 from Tyr. There are 2 Phe, 1 Trp, and 3 Tyr residues in the primary structure of GB1, implying a total of 15 carbon peaks present at natural abundance that share the same ICS within the linewidth of the Phe C*ζ*. The second sideband of the C*ζ* peak overlaps partially with the following aliphatic groups: C*β* from Ala, C*γ*1/2 from Val, C*γ*2 from Ile and Thr, C*δ*1/2 from Leu, and C*ε* from Met. Based on the chemical shifts deposited in the BMRB (entry 7280) for the solution structure of GB1 (PDB: 00002J52), we find 24 overlapping peaks at the second sideband resonance assuming a similar 5 ppm linewidth for these sites. Since this deposition did not include aromatic sites, we used the BMRB chemical shift database for aromatic sites as mentioned earlier. At 1.1% natural abundance, the 43 sites cumulatively behave akin to 0.473 spins, and therefore, the peak evaluated for the CODEX decay should equilibrate at ≈0.404 after correction, which matches the simulated curve (Figure 2C).

The present analysis considers a single, global, *T*_1_ relaxation time for all ^13^C sites. Note that a fast‐relaxing site, like a methyl group, can act as a relaxation sink, decreasing both *S* and *S*_0_. Therefore, when such a site is ^13^C due to natural abundance, the overall contribution to the ensemble averaged CODEX curve is expected to be less. Since the PDSD curve is expected to be similarly affected, we can safely ignore this for the corrected CODEX curve.

An important consideration for the PDSD‐based correction is the inclusion of off‐diagonal peaks in the sideband manifold of the labeled site. CODEX data are often acquired with the spinning frequency kept low for improved PDSD rates. Since different crystallite orientations contribute differently to the centerband and sideband intensities, any magnetization exchange between them will result in crosspeaks. The change in crystallite orientation due to the rotor phase, represented by the *γ*‐angle, also appears as a pseudoexchange crosspeak between *t*_1_ and *t*_2_ even in the absence of magnetization exchange.^[^ ^24^ ^]^ Furthermore, short mixing periods can give phase‐twist lineshapes in a frequency discriminated 2D PDSD spectrum depending on the +1 and −1 quantum coherences acquired in the indirect dimension. A general strategy to eliminate the phase‐twist lineshape is to rotor‐synchronize *t*_m_ for the −1 and *t*_m_ + *t*_1_ for the +1 coherences, respectively.^[^ ^25^, ^26^ ^]^ Suitable isolated ^13^C labeling avoids the need for very short mixing times, minimizing the impact of such effects.

**Figure 3:**
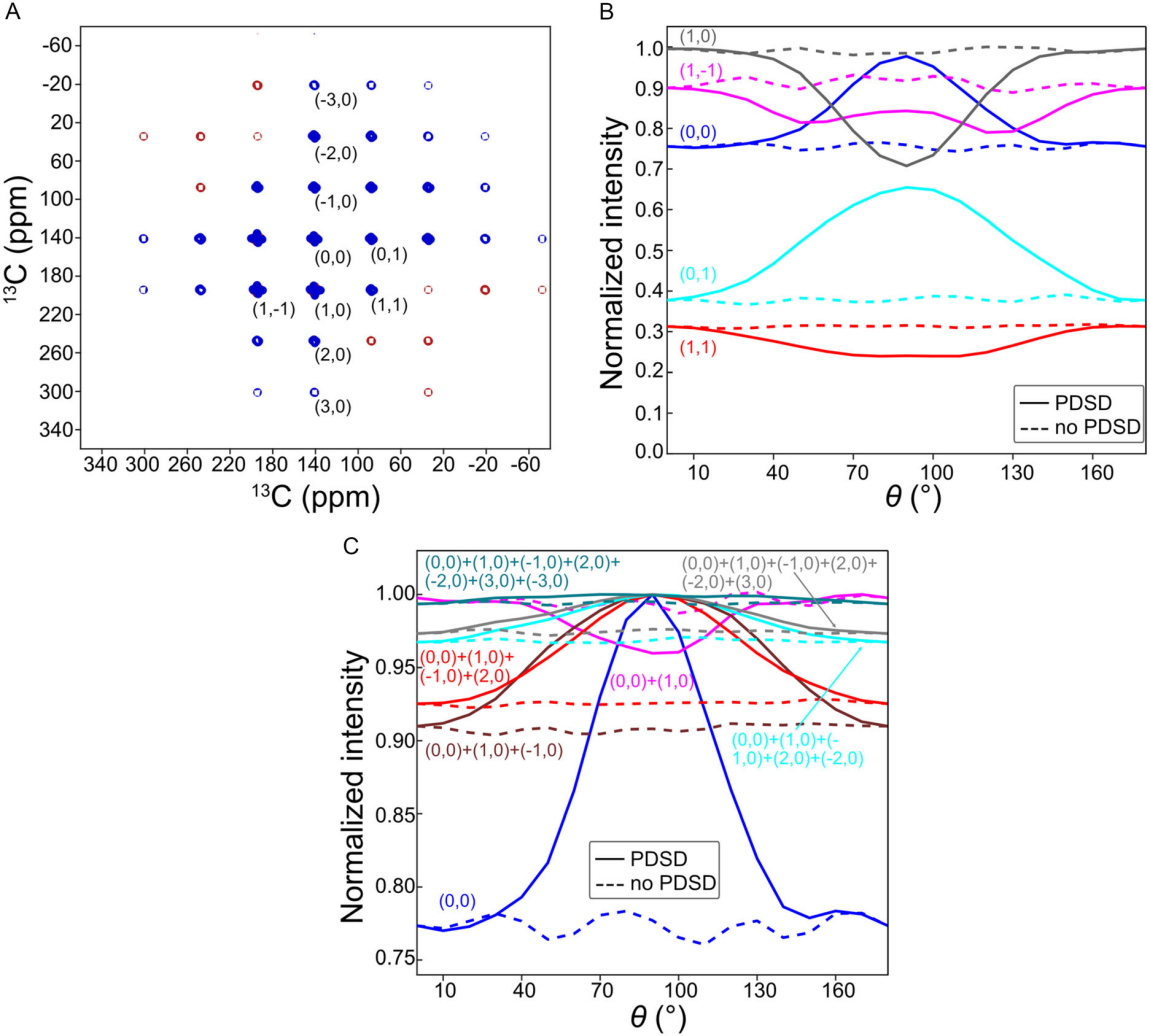
A) Simulated PDSD spectrum with complete magnetization exchange (long mixing). (B) Plot showing the dependence of peak intensity on the relative orientation between the largest axis of the CSA tensors. Data in B) are normalized to the maximum intensity of the strongest peak, the (1,0) peak. C) Plot showing the sum of signal in selected peaks of the sideband manifold for both no mixing (dashed lines) and with complete equilibration (equal spin operator population at the two sites). Each set of selected peaks is normalized to the maximum intensity for that set.

Since we are interested in using the decay of the labeled peak intensity in the PDSD spectrum as a reference, we must account for the intensity distribution across the sideband manifold. **Figure** **3**A shows a simulated PDSD spectrum involving two spins. Two simulations were run, one at a negligible mixing time resulting in no magnetization exchange and the second one with a long mixing time with complete magnetization exchange. The normalized intensities of selected lines in the sideband manifold for the two cases are shown in Figure 3B. There is a clear dependence on the angle between the largest component of the CSA tensor of the two sites (equivalently, due to a change in the rotor phase). As shown in Figure 3C, the difference in intensities of the peaks with and without mixing can be reduced by summing up the intensities in the ω_1_ dimension, i.e., a vertical strip at the isotropic frequency in ω_1_, which we refer to as the minimum set of autocorrelation peaks. The use of the autocorrelation peaks allows us to record the experiments without implementing the rotor synchronized *t*_m_ and *t*_m_ + *t*_1_ scheme.

The correction worked equally well with the diagonal peak alone as with the minimum set of autocorrelation peaks. This resembles the case when the relative orientations of the largest CSA tensor components of the two sites are less than 40° (Figure 3C). We were unable to find information on the specific CSA tensor orientation of the Phe C*ζ* with respect to its molecular geometry, but the largest axis of the tensor can generally be expected to lie perpendicular to the plane of the aromatic ring.^[^ ^27^, ^28^ ^]^ Thus, the sideband intensities are consistent with a relative Phe aromatic ring orientation below about 40° in the GB1 core under the slow freezing conditions of DNP, which is similar to the solution structure of GB1 (PDB: 00002J52), but not consistent with the crystal structure (PDB: 00002QMT).

**Figure 4:**
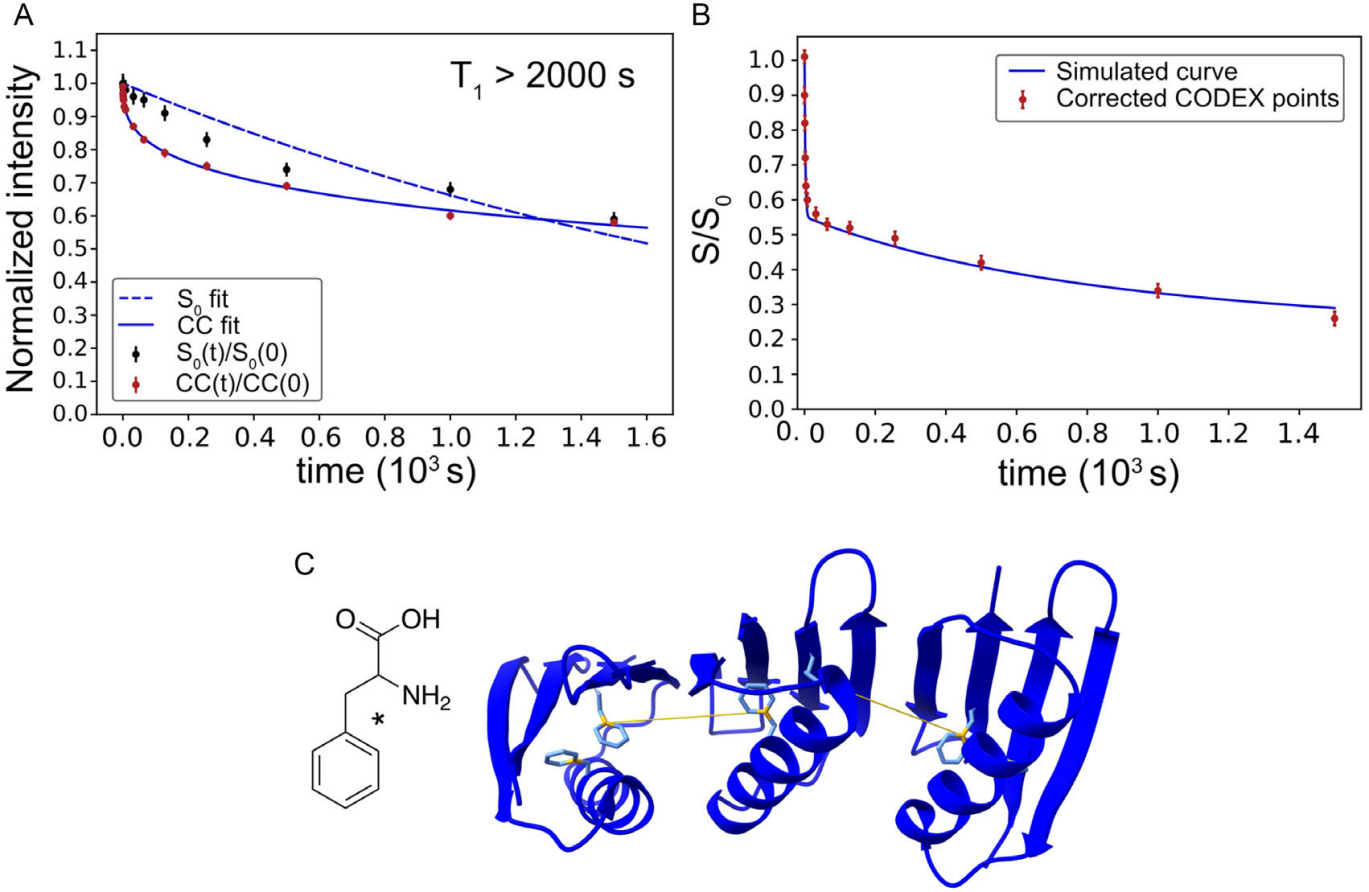
^13^C CODEX of Phe Cγ‐labeled GB1 A) Normalized reference curves, namely, 1D *S*_0_ and 2D autocorrelation peaks. The *S*_0_ decay is fit to an exponential function, and the PDSD decay is fit to a stretched exponential function. The *T*_1_ is estimated to be longer than 2000 s from the *S*_0_ curve, which fit to 2500 s. Note that the low quality of this fit suggests that there may be multiple components with different *T*_1_ values represented in the curve. A possible physical explanation is a faster apparent relaxation time for cases with ^13^C‐labeled methyl groups from the depleted background spins coupled to the labeled site via spin diffusion. This scenario would also explain why the curves appear to converge at the longest mixing times. B) Experimental data and simulated curves with depleted background (0.1%). The solid blue line shows the ensemble average of individual simulation runs. C) The C*γ* position on the Phe ring is highlighted with an asterisk. The structure of three GB1 (PDB: 00002QMT) molecules placed next to each other used for the simulations is shown in blue. The ^13^C‐labeled C*γ* site is marked in gold and the shortest intermolecular spin–spin distance is also displayed in gold.

Having observed the significant effect of background spins on the CODEX decay (Figure 2), we recognized that nearly a two‐fold improvement in sensitivity could be realized if this effect were minimized. We, therefore, included ^13^C‐depleted glucose in the expression medium to lower the background contribution. **Figure** **4** shows data from such a sample of GB1, this time labeled at the *γ* position of Phe with ^13^C. Figure 4A shows how in the absence of the background, the signal survives long enough to extend the mixing time up to 1500 s. Relaxation during the mixing period (^13^C *T*_1_ > 2000 s) is not the primary factor limiting longer mixing times, but rather the long mixing time itself, and the sampling of the 2D spectrum that is used for correction. We carried out the same correction procedure to ensure that the contributions from nonoverlapping background spins are also removed. The impact of the correction was minimal, as shown in Figure 4A. This further obviates the need to estimate overlapping spins since their numbers would be greatly diminished in the total volume across which spin diffusion occurs, unlike in the earlier sample.

Even after background correction, it is evident that the curve decays beyond the expected 0.5 and approaches 0.25 (Figure 4B). For a good signal‐to‐noise ratio (SNR), we prepared a 20% w/v GB1 (38 mM) solution in DNP juice. The highly concentrated sample potentially induces clustering of the protein molecules. We modeled this clustering with simulations of three molecules of GB1 that were manually placed close to each other using ChimeraX, as shown in Figure 4C, such that the shortest distance between a labeled site between the two molecules is about 14 Å. The six‐spin example is an artificial case. There may, for example be more spins present in the vicinity at a potentially greater distance, but collectively providing an effective distance of 14 Å.

The observed decay appears to have two time constants, an initial fast decay followed by a slower one. The initial decay fits the expected distance in GB1 with an *F*(0) value of 4.4 μs, while the latter part of the curve separately fit to an *F*(0) of 8.0 μs for the hypothetical distances of Figure 4C. *F*(0) depends on relative orientations, and for the longer time points in the data, it represents an average quantity for unknown distances and orientations. The final curve was simulated using both *F*(0) values, one for the shorter distance (4.4 μs) and one for the longer distance (8.0 μs). The simulated curve has a reduced *χ* ^2^ of 2.70.

The rapid decay observed in the initial part equilibrated at ≈0.55, which is slightly higher than the expected 0.5. This was accounted for in the simulations by assuming a labeling efficiency of 90%.

We expect DNP‐enhanced ^13^C CODEX to be especially useful in CODEX experiments involving membrane proteins reconstituted in a lipid bilayer. The spins from lipid acyl chain carbons can come close to labeled sites in the protein, especially if the labeled sites are in the transmembrane domain and are lipid facing. For saturated lipids, these acyl chain carbons are well separated from the aromatic region of the spectrum, with prominent peaks in the carbonyl and aliphatic regions. Labeling of the amino acid at the carbonyl position, although favorable for the magnitude of CSA, will overlap with the peaks from conventional ester group containing lipid headgroups. Contribution of overlapping peaks from protein carbonyl spins can be estimated with confidence when the structure is known. By contrast, the unknown lipid headgroup density around the transmembrane region is expected to make it more difficult to accurately estimate the contributions from these headgroups. Therefore, choosing an aromatic site for ^13^C labeling becomes an attractive alternative to ^19^F labeling, as demonstrated here for the model system GB1.

While choosing a spinning frequency for CODEX, it is prudent to ensure that a sideband from an unlabeled site like glycerol in the DNP glassy matrix does not overlap with the centerband or the sidebands of the labeled site. The choice of spinning frequency for CODEX is governed by three considerations: It must be fast enough that the linewidth of the peak is narrowed, slow enough that the spin diffusion rate is still competitive relative to the *R*_1_ relaxation rate to extend the distance range of the technique, and lastly, optimized for the position of the sidebands.

Another technical consideration is the error estimate of the corrected CODEX curve. Predictably, the signal to noise worsens at the longer time points due to signal loss from relaxation and signal decay. Although there is a contribution of error due to the noise from S(t), S(0), CC(t), and CC(0), the combined error is dominated by the noise from S(t). It is therefore advantageous to allocate substantial experimental time to signal averaging of the CODEX dephased curve to reduce errors.

By implementing background correction and combining with ^13^C‐depletion, ^13^C‐based CODEX can become a powerful complement to existing methods of oligomer determination for membrane proteins. Size exclusion chromatography columns, analytical ultracentrifugation, and negative‐stain transmission electron microscopy have been used for oligomer state determination, but entail detergent solubilization of the protein.^[^ ^29^, ^30^ ^]^ Limitations of native mass spectrometry protocols can include the use of supercharging reagents that can significantly perturb the sample and the use of membrane mimetics like amphipols.^[^ ^31^ ^]^ Cross‐linking, followed by sodium dodecyl sulfate‐polyacrylamide gel electrophoresis (SDS‐PAGE) for membrane proteins, is also potentially denaturing and complicated by anomalous migration on the gel caused by differential detergent (and lipid) binding. Mass photometry is a relatively novel technique that can be used for oligomer state determination, but since it detects overall particle size, interpretation for membrane proteins has to consider additional components, such as nanodisks or amphipols.^[^ ^29^ ^]^ The primary advantage of CODEX is that it can be implemented in liposome samples as compared to the aforementioned techniques. CODEX is especially useful for oligomer state determination of small membrane proteins which are below the SNR limit for cryogenic EM and have weak intermolecular interactions, thus making them further unsuitable for mass spectrometry studies.

## 4 Conclusions

The use of CODEX in biological samples enables the determination of the oligomeric state of proteins, but ^13^C CODEX was limited due to the twin challenges of low sensitivity and interference from background spins. To deal with the first challenge, we propose the use of low temperatures combined with DNP to enhance the initial magnetization, thus extending the feasible CODEX mixing time and allowing spin polarization exchange over long distances. To address the second challenge, we propose a 2D PDSD experiment utilizing the same mixing as the CODEX mixing, to quantitate the effect of spin diffusion to peaks with different isotropic chemical shifts. The equilibrium CODEX value which deviates from the expected 0.5 can be rescued using the correction from the 2D PDSD spectrum. The use of ^13^C‐depleted glucose can further decrease the influence of background spins and also permit CODEX measurements up to 1500 s due to the cryogenic conditions and DNP enhancement. We expect that this approach will be beneficial for oligomer state determination of membrane proteins in a lipid bilayer context using ^13^C as a probe.

## 5 Experimental Section

5.1

5.1.1

##### Sample Preparation

GB1 protein was expressed and purified from *E. coli* grown in minimal media according to the published protocol,^[^ ^32^ ^]^ with ^13^C‐*ζ*‐Phe (50 mg L^−1^ minimal medium, FB Reagents) added 30 min before induction. A 48 mM (1.2 mg in 5 μL) GB1 protein solution was prepared in 60% (vol) glycerol (d8, ^13^C‐depleted, Cambridge Isotope Laboratories). Lastly, the polarizing agent, 10 mM TEMPTriPOL‐1, was added to the solution as a lyophilized powder.^[^ ^33^ ^]^

The ^13^C‐*γ*‐Phe GB1 was prepared with the same protocol as mentioned above, but using 0.1% ^13^C‐glucose (^13^C‐depleted glucose) and ^13^C‐*γ*‐Phe (Sigma‐Aldrich). The protein solution was 38 mM (7 mg in 30 μL), and ^13^C‐depleted glycerol (Cambridge Isotope Laboratories) was used.

##### NMR

The NMR experiments were carried out on a Bruker 600 MHz DNP magnet with an HCN probe. The sample was filled inside a thick‐walled 3.2 mm ZrO_2_ rotor with silica plugs placed above and below the sample. The sample temperature was set to 95 K with 1200 liters per hour flow of N_2_ gas, the bearing was set to 98 K, and the drive was set to 105 K. The magic‐angle spinning frequency was set to 8 kHz.

The carbon 90° pulse was carefully optimized since CODEX relies on accurate *π*‐pulses for CSA recoupling. The carbon offset was set to 100 ppm, and the proton offset was set to 4 ppm. For CP, the power on the carbon channel was set to 72.2 kHz and the proton channel power was ramped from 75.9 kHz to 84.3 kHz. The mixing time (*t*_z_) used for the reference experiment was set to 10 ms, and the sum of *t*_m_ and *t*_z_ in the experiment was constant in both CODEX dephased and reference spectra. Each encoding and decoding block was 375 μs long. 89.3 kHz Spinal‐64 was used for proton decoupling during the carbon CSA recoupling block and acquisition. A recycle delay of 7.7 s was used between each scan. An interleaved version of the pulse sequence was implemented where alternate scans of reference and dephased spectra were acquired, which can decrease the influence of longer‐term fluctuations. For example, a slow change in the DNP performance would have minimal influence on the ratio of *S* and *S*_0_ in the interleaved dataset. The phase cycle was set such that only the cosine component of the signal was acquired, such that the relaxation‐induced dipolar‐exchange recoupling (RIDER) effect is minimized.^[^ ^34^, ^35^ ^]^ For the same reason, after CP, a 100 ms z‐filter was implemented and the CSA recoupling time was kept short. The same z‐filter, prior to the encoding block, also served as a premixing period to compensate for variable CP efficiencies and *T*_1_ of various sites across the sample, as previously demonstrated by Xue et al.^[^ ^13^ ^]^ The spinning was stable within ±5 Hz with slightly higher (≈±8 Hz) fluctuation during a liquid *N*_2_ dewar filling event.

The 2D PDSD spectra (Figure 1B) were recorded with the same parameters (CP condition, mixing time, decoupling strength) as the CODEX experiment. The length of the mixing time in the 2D spectrum was kept equal to the total spin diffusion time (*t*_m_ + *t*_z_ in Figure 1B) in the CODEX experiment. In practice, *t*_z_ was short enough that *t*_m_ + *t*_z_ ≈ *t*_m_. The 2D PDSD spectrum did not use an initial pre‐equilibration z‐filter as in the CODEX experiment.

##### CODEX Simulations

We used the exchange matrix approach that was introduced by Mei Hong and coworkers^[^ ^7^ ^]^ to simulate the dephasing observed in GB1. Briefly, the CODEX curve can be simulated classically by constructing a spin diffusion rate matrix that obeys conservation of polarization. The magnetization of a set of spins can then be propagated from time zero to time *t* according to

(2) $$M\left(t\right)=e^{‐\hat{K}}M\left(0\right)$$

(3) $$k_{\text{ij}}=0.5\pi \cdot \omega_{\text{ij}}^{2}\cdot F_{\text{ij}}\left(0\right)$$

(4) $$k_{\text{ii}}=-\sum_{j\neq i}k_{\text{ij}}$$

where ***M*** is a vector representing the magnetization on each spin. The rate matrix $\hat{K}$ is comprised of elements $k_{\text{ij}}$, which represent the rate constant of magnetization exchange between two spins. $k_{\text{ij}}$ depends on the magnitude of the dipolar coupling between the two spins, $\omega_{\text{ij}}$, as well as the overlap integral, $F_{\text{ij}}\left(0\right)$. By design, the rate matrix accounts for both direct and relayed transfers. The overlap integral F(0) represents the probability of zero quantum transitions. In theory, F(0) can be calculated; however, it has conventionally been left as a fit parameter or determined for known model systems. In the past, F(0) was obtained by fitting the CODEX curve for a known crystal form of the same labeled amino acid as that used in the protein. In our case, we left F(0) as a fit parameter, since the internuclear distance in GB1 was known. Note that the value we obtained by fitting has a contribution that arises from relayed transfer through background spins with a different ICS, although this contribution is expected to be small.

At sufficiently long times, the exchange matrix will completely equilibrate the magnetization among the spins and reach a steady‐state value which is a reciprocal of the number of involved sites. The reference spectrum compensates for relaxation losses in the experiment, as mentioned earlier, and relaxation was therefore not included in the simulations.

For CODEX simulations of GB1, the initial magnetization was on the C*ζ* spins of the two phenylalanine residues. Atomic coordinates were taken from the GB1 crystal structure (PDB: 00002QMT). To account for the presence of natural abundance ^13^C spins, additional spins were included in the simulation according to the GB1 crystal structure with a 1.1% probability. Magnetization was then allowed to equilibrate using the rate matrix approach mentioned above. The distances between the spins were calculated from the positions of the atoms in the PDB file, and these distances were used to calculate the dipolar coupling constants between the spins. For simplicity, a single F(0) value was used for both the labeled and natural abundance sites. The best fit F(0) value was determined to within 0.1 μs by averaging 300 simulations for each test F(0) value and selecting the F(0) with the lowest reduced *χ* ^2^ with respect to the experimental data. Subsequently, this F(0) was used to run 1000 individual simulations for the conditions described in the Results and Discussion. The Python code used for these simulations is provided in the Supporting Information.

The same simulation protocol was applied for the C*γ*‐labeled GB1 case, without any background spins. The simulation was compared to the corrected CODEX curve. The simulation included 90% labeling efficiency at the labeled site. Two different *F*(0) values were used as explained in the main text, one for shorter distances and one for longer distances.

##### PDSD Simulations

A two‐spin simulation was set up with identical ICS, but variable relative orientations between the two spins. Powder averaging was implemented with 538 orientations that related the molecular and rotor frames. The spinning was 8 kHz, and the dwell time was 12.5 μs. The signal was apodized with a monoexponential function with a time constant of 2.5 ms before Fourier transformation. As per the Haeberlen convention, the *δ*_CSA_ was 18.75 kHz and *η* was 0.81, which were determined for the Phe C*ζ* site from experimental data using the SOlid Lineshape Analysis (SOLA) module within Bruker's TopSpin software. For the C*γ* site, *δ*_CSA_ was fit to 19.35 kHz, with *η* of 0.5. The MATLAB code used for the simulation is provided in the Supporting Information.

## Conflict of Interest

The authors declare no conflict of interest.

## Supporting information

Supplementary Material

## Data Availability

The data that support the findings of this study are available from the corresponding author upon reasonable request.
