# Supplementary material for: Carbon‐13 Centerband‐Only Detection of EXchange with Dynamic Nuclear Polarization
Source: Chemphyschem. 2026 Jan 30;27(2):e202500585. doi: 10.1002/cphc.202500585 (PMC12856727; doi:10.1002/cphc.202500585)
Supplement: Supplementary file 1 — Supplementary Material [file CPHC-27-e202500585-s001.pdf]

```
from random_13C import random_13C
import numpy as np
import matplotlib.pyplot as plt
from datetime import datetime

start_time = datetime.now() # track code time

'''DATA'''
t_exp = np.array([0, 0.512, 1, 2, 4, 8, 32, 64, 128, 256, 500, 1000, 1500],
                 dtype=int)

CODEX_corr = np.array([1.01, 0.90, 0.82, 0.72, 0.64, 0.60, 0.56, 0.53, 0.52,
                       0.49, 0.42,
                       0.34, 0.26], dtype=float)

CODEX_corr_err = np.array([0.0184, 0.0230, 0.0217, 0.0179, 0.0193, 0.0192,
                           0.0182, 0.0159, 0.0169, 0.0192, 0.0199, 0.0192,
                           0.0195], dtype=float)

'''FILE PREPARATION'''
# Prepare the input file by deleting all the lines from the pdb files except
# the required atom coordinates
# To do this: first, selct the required atoms in Chimera, then invert
# selection, and delete all remaining atoms and save the file as a new pdb file
# Open the new pdb file with a text editor and remove all the additional lines
# without the atom coordinates
# Final file has the following format:
#    ATOMNAME X-COORDINATE Y-COORDINATE Z-COORDINATE

'''INPUT PARAMETERS'''
labeled_site = 'PheCG_6SpinCluster_14Adist.txt'  # Input the labelled site
                                                 # atom coordinates
labeling_efficiency = 0.9
F0 = [2, 15, 0.05]  # Input the [Start, Stop, Step] values for the Overlap
                    # integral
gamma = 6.728284e7  # Gyromagnetic ratio of carbon
uplimit = 1.5e6  # Upper bound of CODEX plot in ms (x axis)
step = 150  # Time increment in ms (smaller equals smoother curves)
runs = 500  # Iterations to select a random population of natural abundance
            # 13C spins from the pdb file
''''''

'''SIMULATION'''
npts = int(uplimit/step)  # Number of points
F0 = np.array(F0, dtype=float)
f0 = np.arange(F0[0], F0[1], F0[2])
f = len(f0)

end_time = datetime.now() # track code time
print('Duration: {}'.format(end_time - start_time)) # track code time

def CODEX(fname1, abundance1, fname2, abundance2):
    n = 0
    while n ==0:        # Ensure no cases of 0 labelled sites since n=0 causes
                        # code error
        C13 = random_13C(labeled_site, labeling_efficiency) # Generate random
                        # population of 13C spins based on labeling efficiency
        n = len(C13)
    atoms1 = random_13C(fname1, abundance1)  # Generate random population of
                                             # 13C spins based on abundance
    atoms2 = random_13C(fname2, abundance2)  # Generate random population of
```

```
                                        # 13C spins based on abundance

from scipy.linalg import expm

mu_0 = 1.25663706212e-6  # Vacuum permeability
hbar = 1.054571817e-34  # Planck constant
ang = 1e-10  # One angstrom
powd = 0.2  # Powder average of angular dependence

if len(atoms1)>0:
    coords = np.vstack((C13, atoms1))
else:
    coords = C13

if len(atoms2)>0:
    coords = np.vstack((coords, atoms2))

l = len(coords)

dismatrix = np.empty([l, l])  # Initialize a distance matrix for all
                              # distances

for i in range(l):
    for j in range(i+1, l):
        x = coords[i, 0] - coords[j, 0]
        y = coords[i, 1] - coords[j, 1]
        z = coords[i, 2] - coords[j, 2]
        d = np.sqrt(x**2+y**2+z**2)
        dismatrix[i,j] = d
        dismatrix[j,i] = d

C_1a = (mu_0 * hbar * gamma**2)/(4*np.pi*ang**3)  # A C-C dip coup (in
                                                  # rads!!)
dipcoup = C_1a
M0matrix = np.identity(l)  # Initial state is identity matrix of
                           # dimensions [l, l
I = np.identity(l)
dis = dismatrix+I  # Remove zeroes along the diagonal before reciprocal
                   # operation
W = (np.reciprocal(dis**3))*dipcoup  # Homonuclear dipolar coupling
                                     # strength
Wsqu = W**2  # Coupling squared

for i in range(l):  # Detailed balance
    Wsqu[i, i] = 0  # Zero the diagonal of the coupling matrix
    Wsqu_sums = np.sum(Wsqu, axis=1)  # Sum each column
    Wsqu[i, i] = -Wsqu_sums[i]  # Diag set to neg sum

# Calculate the exchange matrix K
K = 0.5*np.pi*Wsqu*powd/1000000
K = np.kron(f0, K)  # Kronecker product of each F0 value with constants to
                    # get K for each F0
#K has the shape (n, n*f)
# K matrix has to be reshaped into square sub-matrices for each F0 to
# exponentiate the matrix
K_F0 = np.empty([f, l, l])
idx = 0
for i in range(f):
    K_sub = np.stack(K[:, idx:(idx+l)])
    idx += l
    K_F0[i, :, :] = K_sub
```

```
    # Mt is a 4d matrix
    # 1st axis is F0 values
    # 2nd axis is the ending spin
    # 3rd axis is the starting spin
    # 4th axis is time
    Mt = np.empty([f, l, l, npts])
    prop = np.empty([f, l, l])

    for i in range(f):
        prop[i] = expm(step/1000*K_F0[i])

    for currSpin in range(l):
        # For each spin,
        # calc the dip coup matrix W and the dip coup square
        M0 = M0matrix[:, currSpin]  # Extract the vector for init mag on spin
        for i in range(f):
            currMat = expm(0/1000*K_F0[i])  # Calculate the initial SD matrix
            t_idx = 0  # Initialize a time index
            for t in np.arange(0, uplimit, step):
                # For each time step, calculate exchange process
                Mt[i, currSpin, :, t_idx] = np.matmul(currMat, M0)  # Calculate
                                                                     # Mt
                currMat = np.matmul(prop[i], currMat)  # Increment exchange
                                                       # matrix
                t_idx = t_idx+1  # Increment time index
            #end_time = datetime.now() # track code time
            #print('Duration: {}'.format(end_time - start_time)) # track code
                                                                 # time


    Mt_avg = np.empty([f, 2, npts])  # Initialize a matrix for avg
                                     # magnetization
    Mt_avg[:, 0, :] = np.arange(0, uplimit, step)  # Second row is time, third
                                                   # row is M(t) avg

    # Calc avg mag at each time pt for each F0 only for the labelled spins
    for i in range(n):
        Mt_avg[:, 1, :] = np.trace(Mt[:, :n, :n, :], axis1=1, axis2=2)

    sim = Mt_avg[:, 1, :]

    return sim

curves = np.empty([runs, f, npts])

'''INPUT PARAMETERS'''
for i in range(runs):
    # Include (1) file name of sites without labelling and (2) expected
    # abundance
    curves[i, :, :] = CODEX('PheCG_4SpinCluster_15Adist.txt', 0,
                            'PheCG_4SpinCluster_15Adist.txt', 0)  # CODEX
                                                                  # curve with background
    end_time = datetime.now() # track code time
    print('Duration: {}'.format(end_time - start_time)) # track code time
    print('Runs completed: '+str(i+1))
''''''

'''SIMULATION'''
all_curves = np.sum(curves, axis=0)/runs  # Take average over all the curves
                                          # in a given number of runs
```

```
curve_normalized = all_curves[:, :]/all_curves[:, 0:1]
time_ax = np.arange(0, uplimit, step)

'''F(0) FITTING'''
chi2 = np.empty(f) # Initiate empty list for chi square test
for i in range(f):
    # Calculate the y-distance of each point to the curve
    y_dist_to_curve = np.interp(t_exp, time_ax/1000, curve_normalized[i, :])
    # Chi square test formula
    chi2[i] = np.sum(((CODEX_corr-y_dist_to_curve)**2)/CODEX_corr_err**2)
v = len(CODEX_corr)-1  # v is the degrees of freedom
chi2_red = chi2/v
min_chi2_red = min(chi2_red)  # Minimum reduced chi square
chi2_red_index = (np.where(chi2_red == min_chi2_red)[0])  # Find the array
                                                    # index number of the X2
F0_fit = f0[chi2_red_index]  # Use the same index number to select the
                             # corresponding F0 value
print('Fit F0 = ' + str(F0_fit))
print('Reduced chi square = ' + str(min_chi2_red))
CODEX_sim = curve_normalized[chi2_red_index, :].squeeze()
''''''

'''PLOTTING PARAMETERS'''
plt.figure(dpi=1000)
plt.plot(time_ax/1000, CODEX_sim, color='mediumblue',
         label='F(0) best fit curve', linestyle='-', linewidth=3)
plt.errorbar(t_exp, CODEX_corr, yerr=CODEX_corr_err, fmt='.',
             color='firebrick', label = 'Corrected CODEX points',
             linestyle='None', capsize=1)
plt.legend(loc='upper right')
plt.yticks(np.arange(0, 1.1, 0.05))
plt.xlim(-20, None)
plt.xlabel('t (s)')
plt.ylabel('S/S$^o$')
plt.title('CG-Phe 2QMT 6-spin cluster model')
plt.savefig('CODEX_2QMT_PheCG_6cluster_2.ps')
plt.savefig('CODEX_2QMT_PheCG_6cluster_2.pdf')
plt.show()

plt.figure(dpi=1000)
plt.plot(f0, chi2_red, color='mediumblue', label='F(0) reduced chi square',
         linestyle='-', linewidth=3)
plt.xlabel('F(0) ($\mu$s)')
plt.ylabel('$\chi$$^2$$_red$')
plt.ylim(0, None)
plt.legend(loc='upper right')
plt.title('CG-Phe 2QMT 6-spin cluster')
plt.savefig('redX2_2QMT_PheCG_6cluster_2.ps')
plt.savefig('redX2_2QMT_PheCG_6cluster_2.pdf')
plt.show()

end_time = datetime.now() # track code time
print('Duration: {}'.format(end_time - start_time)) # track code time
```

MATLAB code for PDSD simulation:

```
clearvars;
clf;

A=dlmread('538.dat');      % number of the orientations
Siz=length(A);

%%%%%%%%%%    INPUT PARAMETERS   %%%%%%%%%%%%
v_R = 8;                              %   spinning speed in kHz

CSA = 18.75;                          %   chemical shift anisotropy in [kHz]
eta_CSA = 0.81;                       %   anisotropy parameter

kappa = 1;                            %   the exchange rate in [Hz]
D6_max = 5;                           %   the mixing time for exchange in [s]


%%%%%%%%%%%        END     %%%%%%%%%%%%%%%%%%%


Tr = 1/v_R;  %rotor period in ms
I=[1,0;0,1];
Iz=0.5*[1,0;0,-1];
Ix=0.5*[0,1;1,0];
Iy=0.5*[0,-1i;1i,0];
Norm = trace(Ix*Ix);
dim = length(I);

T_2eff = 2.5;   % in ms
T2eff = T_2eff/Tr;
dt_t1_t2=0.1;

CSA_N = -2*pi*CSA/v_R;
p20 = 1;
p22 = (2/3)^(0.5)*eta_CSA/2;




Ntot = 10/Tr;
NN=1;
N_Tr = 1/dt_t1_t2;
t_tot = (0:dt_t1_t2:Ntot)';


v_L=0.15;
CF = 139;
ISO = 141;
OFF_H = -(ISO - CF)*v_L;
p_offset = OFF_H/v_R;

side_band = v_R/v_L;
```

```
t_mix_kappa = D6_max;
for aa=1:1
    ax11_long=0.5*(1 + exp(-t_mix_kappa(aa,1)*kappa));
    ax12_long=0.5*(1 - exp(-t_mix_kappa(aa,1)*kappa));
end

D6_max = 0.0000002;                     %   the mixing time for exchange in [s]
t_mix_kappa = D6_max;
for aa=1:1
    ax11_short=0.5*(1 + exp(-t_mix_kappa(aa,1)*kappa));
    ax12_short=0.5*(1 - exp(-t_mix_kappa(aa,1)*kappa));
end

for AA=1:19
    beta_orient = 10*(AA-1);                %   the orientation between two CSA interactions↙
in (°)
    St1_t2_long = 0;
    St1_long = 0;

    St1_t2_short = 0;
    St1_short = 0;
for l=1:length(A)
    DT_M = 0;
    for ad=1:N_Tr
        V_t1_S1=eye([dim dim]);
        dt = dt_t1_t2/NN;
        for af=1:NN
            ti=DT_M+(af-1)*dt;
            tf=DT_M+af*dt;
            for sc=1:1
                VD(sc,1)=-Orient(A(l,1),A(l,2),A(l,3),0,0,0,ti,tf,p20(sc,1),p22(sc,1));
            end
            H = CSA_N*VD(1,1)*Iz+2*pi*dt*p_offset*Iz;
            [V1,E]=eig(H);
            Eh=eye([dim dim]);
            for ss=1:dim
                Eh(ss,ss)=exp(-1i*E(ss,ss));
            end
            H1=V1*Eh/V1;
            V_t1_S1=H1*V_t1_S1;
        end
        VT_S1{ad} = V_t1_S1;
        DT_M = DT_M + dt_t1_t2;
    end
    U_t_b = eye([dim dim]);
    ii = 2;
    for aa=1:Ntot
        for ab=1:N_Tr
            U_t_b = VT_S1{ab}*U_t_b;
            pho_b = U_t_b*Ix/U_t_b;
```

```
            Sig_bx(ii,1)=(real(trace(pho_b*Ix))/Norm);
            Sig_by(ii,1)=(real(trace(pho_b*Iy))/Norm);
            ii = ii + 1;
        end
    end
    Sig_bx(1,1) = 1;
    Sig_by(1,1) = 0;
    Sa = (Sig_bx - 1i*Sig_by).*exp(-t_tot/T2eff);

    DT_M = 0;
    for ad=1:N_Tr
        V_t1_S1=eye([dim dim]);
        dt = dt_t1_t2/NN;
        for af=1:NN
            ti=DT_M+(af-1)*dt;
            tf=DT_M+af*dt;
            for sc=1:1
                VD(sc,1)=-Orient(A(l,1),A(l,2),A(l,3),0,beta_orient*pi/180,0,ti,tf,p20↙
(sc,1),p22(sc,1));
            end
            H = CSA_N*VD(1,1)*Iz+2*pi*dt*p_offset*Iz;
            [V1,E]=eig(H);
            Eh=eye([dim dim]);
            for ss=1:dim
                Eh(ss,ss)=exp(-1i*E(ss,ss));
            end
            H1=V1*Eh/V1;
            V_t1_S1=H1*V_t1_S1;
        end
        VT_S1{ad} = V_t1_S1;
        DT_M = DT_M + dt_t1_t2;
    end
    U_t_b = eye([dim dim]);
    ii = 2;
    for aa=1:Ntot
        for ab=1:N_Tr
            U_t_b = VT_S1{ab}*U_t_b;
            pho_b = U_t_b*Ix/U_t_b;
            Sig_bx(ii,1)=(real(trace(pho_b*Ix))/Norm);
            Sig_by(ii,1)=(real(trace(pho_b*Iy))/Norm);
            ii = ii + 1;
        end
    end
    Sig_bx(1,1) = 1;
    Sig_by(1,1) = 0;
    Sb = (Sig_bx - 1i*Sig_by).*exp(-t_tot/T2eff);
    for aa=1:length(Sa)
        for ab=1:length(Sb)
            Sab_cryst(aa,ab) = ax11_long*(Sa(ab,1)*Sa(aa,1)+Sb(ab,1)*Sb(aa,1))+ax12_long*↙
(Sa(ab,1)*Sb(aa,1)+Sb(ab,1)*Sa(aa,1));
        end
```

```
    end
    St1_t2_long = St1_t2_long + 0.5*Sab_cryst*A(l,4);
    St1_long = St1_long + Sb*A(l,4);

    for aa=1:length(Sa)
        for ab=1:length(Sb)
            Sab_cryst(aa,ab) = ax11_short*(Sa(ab,1)*Sa(aa,1)+Sb(ab,1)*Sb(aa,1))↙
+ax12_short*(Sa(ab,1)*Sb(aa,1)+Sb(ab,1)*Sa(aa,1));
        end
    end
    St1_t2_short = St1_t2_short + 0.5*Sab_cryst*A(l,4);
    St1_short = St1_short + Sb*A(l,4);
end


dt_F = dt_t1_t2/v_R;
fs= (1/dt_F);
ii_N = 1;
X_ii = length(Sa);
while X_ii > 1
    ii_N = ii_N + 1;
    X_ii = length(Sa) /(2^ii_N);
end

FT_2D_long = (fft2(St1_t2_long,2^ii_N,2^ii_N));


m = length(FT_2D_long(1,:));           % Window length
n = pow2(nextpow2(m));  % Transform length
f0 = ((-n/2:n/2-1)*(fs/n))';  % 0-centered frequency range
Fo = f0/v_L+CF;

[~,ds(1,1)] = min(abs(Fo - ISO));
[~,ds(2,1)] = min(abs(Fo - (ISO + side_band)));
[~,ds(3,1)] = min(abs(Fo - (ISO - side_band)));

[~,ds(4,1)] = min(abs(Fo - (ISO + 2*side_band)));
[~,ds(5,1)] = min(abs(Fo - (ISO - 2*side_band)));

[~,ds(6,1)] = min(abs(Fo - (ISO + 3*side_band)));
[~,ds(7,1)] = min(abs(Fo - (ISO - 3*side_band)));

y0= abs(fftshift(FT_2D_long));           % Rearrange y values
max_max = max(max(y0));
%y0 = y0/max_max;

XY = [ds(1,1), ds(1,1); ds(2,1), ds(2,1); ds(1,1), ds(2,1);ds(3,1), ds(2,1);ds(3,1), ds↙
(1,1);];
for ab = 1:length(XY(:,1))
    Diag_sid_D6_long(AA,ab) = max(max(y0(XY(ab,1) - 8:XY(ab,1) + 8,XY(ab,2) - 8:XY(ab,2)↙
+ 8)));
```

```
end

vert_XY = [ds(1,1), ds(1,1);ds(1,1), ds(2,1);ds(1,1), ds(3,1);ds(1,1), ds(4,1);ds(1,1),↙
ds(5,1);ds(1,1), ds(6,1);ds(1,1), ds(7,1);];
for ab = 1:length(vert_XY(:,1))
    vert_sid_D6_long(AA,ab) = max(max(y0(vert_XY(ab,1) - 8:vert_XY(ab,1) + 8,vert_XY(ab,↙
2) - 8:vert_XY(ab,2) + 8)));
end

goriz_XY = [ds(1,1), ds(1,1);ds(2,1), ds(1,1);ds(3,1), ds(1,1);ds(4,1), ds(1,1);ds(5,1),↙
ds(1,1);ds(6,1), ds(1,1);ds(7,1), ds(1,1);];
for ab = 1:length(goriz_XY(:,1))
    goriz_sid_D6_long(AA,ab) = max(max(y0(goriz_XY(ab,1) - 8:goriz_XY(ab,1) + 8,goriz_XY↙
(ab,2) - 8:goriz_XY(ab,2) + 8)));
end

FT_2D_short = (fft2(St1_t2_short,2^ii_N,2^ii_N));


m = length(FT_2D_short(1,:));           % Window length
n = pow2(nextpow2(m));  % Transform length
f0 = ((-n/2:n/2-1)*(fs/n))';  % 0-centered frequency range
Fo = f0/v_L+CF;

y0_short= abs(fftshift(FT_2D_short));          % Rearrange y values

%y0_short = y0_short/max_max;

for ab = 1:1:length(XY(:,1))
    Diag_sid_D6_short(AA,ab) = max(max(y0_short(XY(ab,1) - 8:XY(ab,1) + 8,XY(ab,2) - 8:XY↙
(ab,2) + 8)));
end


for ab = 1:length(vert_XY(:,1))
    vert_sid_D6_short(AA,ab) = max(max(y0_short(vert_XY(ab,1) - 8:vert_XY(ab,1) + 8,↙
vert_XY(ab,2) - 8:vert_XY(ab,2) + 8)));
end

for ab = 1:length(goriz_XY(:,1))
    goriz_sid_D6_short(AA,ab) = max(max(y0_short(goriz_XY(ab,1) - 8:goriz_XY(ab,1) + 8,↙
goriz_XY(ab,2) - 8:goriz_XY(ab,2) + 8)));
end

end
```
